# Transgenic Silkworm-Based Silk Gland Bioreactor for Large Scale Production of Bioactive Human Platelet-Derived Growth Factor (PDGF-BB) in Silk Cocoons

**DOI:** 10.3390/ijms19092533

**Published:** 2018-08-27

**Authors:** Wenjing Chen, Feng Wang, Chi Tian, Yuancheng Wang, Sheng Xu, Riyuan Wang, Kai Hou, Ping Zhao, Ling Yu, Zhisong Lu, Qingyou Xia

**Affiliations:** 1 State Key Laboratory of Silkworm Genome Biology, Southwest University, Chongqing 400715, China; chenwj@mail.sustc.edu.cn (W.C.); wangf1986@swu.edu.cn (F.W.); tianchi528@gmail.com (C.T.); ych.wang1989@gmail.com (Y.W.); xusheng1324@gmail.com (S.X.); rywang2020@gmail.com (R.W.); minyang940429@gmail.com (K.H.); Zhaop@swu.edu.cn (P.Z.); 2 Chongqing Engineering and Technology Research Center for Novel Silk Materials, Southwest University, Chongqing 400715, China; 3 Chongqing Key Laboratory of Mulberry Silkworm, Southwest University, Chongqing 400715, China; 4 Institute for Clean Energy & Advanced Materials, Faculty of Materials & Energy, Southwest University, Chongqing 400715, China; lingyu12@swu.edu.cn (L.Y.); zslu@swu.edu.cn (Z.L.)

**Keywords:** silkworm, silk gland, recombinant expression, human platelet-derived growth factor

## Abstract

Human platelet derived growth factor (PDGF) is a major therapeutic protein with great demand in the clinical setting; however, its rate of supply is far from meeting needs. Here, we provide an effective strategy to produce PDGF-BB in large quantities using a transgenic silkworm. The codon-optimized *PDGF-B* gene regulated by the highly efficient *sericin-1* expression system was integrated into the genome of a silkworm. The high transcriptional expression of the PDGF-BB gene in the transgenic silkworm competitively inhibited the transcription expression of the endogenous *sericin-1* gene which caused a significant 37.5% decline. The PDGF-BB synthesized in the middle silk gland (MSG) of transgenic silkworms could form a homodimer through intermolecular disulfide bonds, which is then secreted into sericin lumen and finally, distributed in the sericin layer of the cocoon. In this study, a protein quantity of approximately 0.33 mg/g was found in the cocoon. Following a purification process, approximately 150.7 μg of recombinant PDGF-BB with a purity of 82% was purified from 1 g of cocoons. Furthermore, the bioactivity assays showed that the purified recombinant PDGF-BB was able to promote the growth, proliferation and migration of NIH/3T3 cells significantly. These results suggest that the silk gland bioreactor can produce active recombinant PDGF-BB as an efficient mitogen and wound healing agent.

## 1. Introduction

Human platelet derived growth factor (PDGF) belongs to the glycoprotein dimer family, and has four isoforms: PDGF-A, PDGF-B, PDGF-C, and PDGF-D [1]. The PDGFs specifically bind to the PDGF receptor (PDGFR) through a homodimer or heterodimer, such as PDGF-AA, PDGF-BB, or PDGF-AB, for molecular signal transition [2]. Among these compounds, PDGF-BB, constructed of two PDGF-B chains is the most commonly found dimer in the human body. The molecular weight of PDGF-BB is 14 kDa, and it plays an important role in many processes, such as cell proliferation and wound healing, due to it is a strong activity as a mitogen for various cell types, especially vascular endothelial cells (VECs) and bone marrow mesenchymal stem cell (BMSCs) [1]. In particular, when angiorrhexis occurs, the platelets can release PDGF-BB to recruit the perivascular cells and vessel smooth muscle cells and to promote VEC proliferation to facilitate vessel remolding. As for bone repairing, PDGF-BB can accelerate the differentiation of BMSCs into osteoblasts which can bring about more osteocytes to achieve bone repair [3]. Thus, PDGF-BB is regarded as a desirable pharmaceutical and was recently approved by the FDA for osteochondritis (OCT) and cardiovascular disorder treatment in the clinical setting. In addition, through innovation in the cosmetics industry, PDGF-BB has also been considered a main ingredient in solving skin problems, including diminishing wrinkles and moisturizing [4]. Thus, the clinical demand for PDGF-BB for a wide range of applications in biomedicine and bio-cosmetics has increased, drawing much attention to the recombinant expression of PDGF-BB. For decades, several attempts have been made to produce the recombinant PDGF-BB in the hosts of the yeasts *Pichia Pink* [5] and tobacco *N. tabacum* L. [6]. However, poor yields, complicated processing, and low bioactivity have severely limited their ability to meet the market’s demand, especially with the increasing number of applications for cell therapy and translational medicine. As a result, constructing an efficient strategy for the cost effective and mass production of recombinant PDGF-BB with native bioactivity is urgent and necessary.

The domesticated silkworm possesses a great ability to synthesize abundant silk proteins within the short term in silk glands and secrete them into silk to make cocoons. Thus, the mass production of recombinant proteins by transgenic silkworms is a desirable bioreactor [7]. Silk proteins mainly consist of fibroin proteins (75%) and sericin proteins (25%) [8]. The fibroin proteins are synthesized by the posterior silk glands cells which consist of fibroin heavy chains (H-chains), fibroin light chains (L-chains), and fibrohexamerins in a molar ratio of 6:6:1 [9]. The sericin proteins are mainly encoded by the *sericin-1*, *sericin-2*, and *sericin-3* genes, respectively [10]. The genetic transformation tool mediated by the *piggyBac* transposon was successfully applied in silkworms in 2000 [11]. The transgenic silkworm has been genetically engineered as an ideal bioreactor to express recombinant foreign proteins along with the synthesis of their silk proteins. Two major expression systems based on the usage of the fibroin and sericin-1 promoters have been successfully constructed [12,13]. Thereafter, more than ten exogenous proteins with various bio-functions and application potential have been successfully expressed in the silk glands of transgenic silkworms and cocoons, including human type III pro-collagen, human serum albumin, human acid fibroblast growth factor, and antibodies [14,15,16]. These efforts have led to the silk glands of transgenic silkworm being very close to the ideal bioreactor organ to massively produce the recombinant pharmaceutical proteins to meet the increased demand of the clinic.

In this study, we provided an effective strategy to produce bioactive recombinant PDGF-BB proteins in silk cocoons of transgenic silkworm using our previous established *sericin-1* expression system [17]. The recombinant PDGF-BB was specifically synthesized into the middle silk gland cells and secreted into the sericin lumen. The transcriptional and translational expression of the recombinant PDGF-BB was well studied. Furthermore, the recombinant PDGF-BB was purified from cocoons with 82% purity and 50.2% recovery rate, and the bioassays further indicated that the purified recombinant PDGF-BB possesses the bio-function to promote NIH/3T3 cell proliferation and migration. The results strongly suggest the potential of silk gland-based bioreactors of silkworm to cost effectively and massively produce these valuable recombinant proteins with natural bioactivity in silk cocoons.

## 2. Results

### 2.1. Generation of the PDGF-BB Transgenic Silkworm

**Figure 1:**
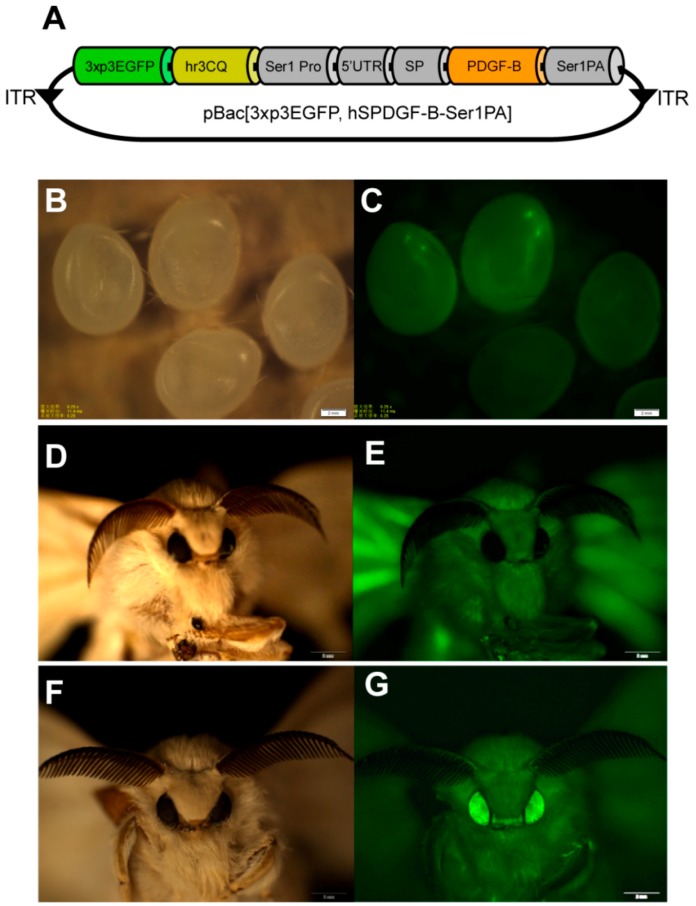
Generation of the PDGF-BB transgenic silkworm. (**A**) Structural map of the transgenic vector. 3xp3EGFP represents the selection marker of the transgene; hr3CQ represents an enhancer isolated from the *Bombyx mori* nuclear polyhedrosis virus (*BmNPV*) genome of a Chongqing stain; Ser1 Pro represents the promoter region of the *sericin-1* gene; SP indicates the signal peptide of the *sericin-1* gene; Ser1PA indicates the polyA sequence of the *sericin-1* gene. (**B**) White light image of a transgenic silkworm egg. (**C**) Fluorescence image of a transgenic silkworm egg. (**D**) White light image of a wild type silkworm moth. (**E**) Fluorescence image of a wild type silkworm moth. (**F**) White light image of a transgenic silkworm moth. (**G**) Fluorescence image of a transgenic silkworm moth. Scale bars are 2 mm.

In previous reports, we established an efficient *sericin-1* expression system to produce the recombinant foreign proteins in the middle silk gland of larval silkworm [17]. In this study, this expression system was applied to the recombinant expression of PDGF-BB by the transgenic silkworm. A *piggyBac*-based transgenic vector, phSPDGFSer1PA, was firstly designed and constructed (Figure 1A). The coding sequence of the *PDGF-B* gene was optimized according to the silkworm codon usage bias to achieve the adaptative and considerable expression of PDGF-BB in the silkworm. An hr3 enhancer enhanced *sericin-1* promoter with its intact signal peptide was used to control the spatiotemporal expression and secretion of the *PDGF-B* gene in the transgenic silkworm, the 3xp3-EGFP [18,19], which could achieve the specific expression of EGFP in the ocelli and compound eyes of silkworm, was used as the genetical screening marker. Ninety-one out of 200 microinjected eggs were hatched and fed carefully until the moth stage for the oviposition of G1 offspring. Five out of 13 detected broods contained different numbers of positive individuals (data not shown) with specific EGFP emission in the ocelli of the body pigmentation stage of the embryos (Figure 1B,C) and the compound eyes of the moths (Figure 1D–G), suggesting the successful transformation of hereditable transgenesis in silkworms. The transformation frequency for the PDGF-BB transgenic silkworm was estimated to be approximately 38%. Subsequently, all of the 3xP3-EGFP positive silkworm individuals were carefully fed, and 25 of them survived to spin silks to build the cocoons. The cocoons from these 25 individuals were collected for further analysis.

### 2.2. The Expression Analysis of the Recombinant PDGF-BB Proteins in Transgenic Silkworm

**Figure 2:**
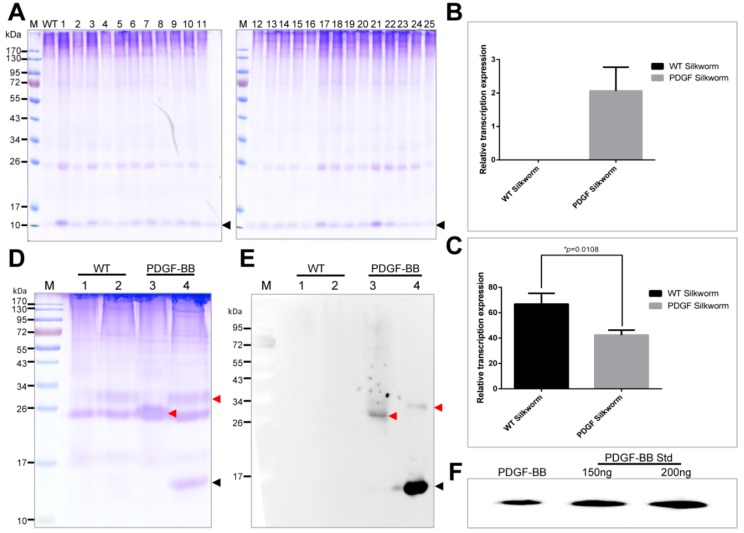
Expression analysis of PDGF-BB in transgenic silkworms. (**A**) SDS-PAGE analysis of the recombinant PDGF-BB protein in the cocoons from the 25 of positive silkworm individuals. The black arrowhead points to the recombinant PDGF-BB. (**B**,**C**) The transcriptional expression analysis of the *PDGF-B* gene and the endogenous *sericin-1* gene between the wild type silkworm and the transgenic silkworm PDGF-21 strain by RT-PCR. All experiments were performed in three biological replicates. Values are represented as the mean ± SE (error bars). For the significance test: * *p* < 0.05 vs. control. (**D**,**E**) SDS-PAGE and Western blot analyses to identify the dimer form of recombinant PDGF-BB synthesized in the cocoons of the transgenic silkworm PDGF-21 strain. The black arrowhead points to the recombinant PDGF-BB monomer; the red arrowhead points to the recombinant PDGF-BB homodimer. (**F**) The content calculation of the recombinant PDGF-BB proteins in the cocoon weight of the transgenic silkworm PDGF-21 strain.

**Figure 3:**
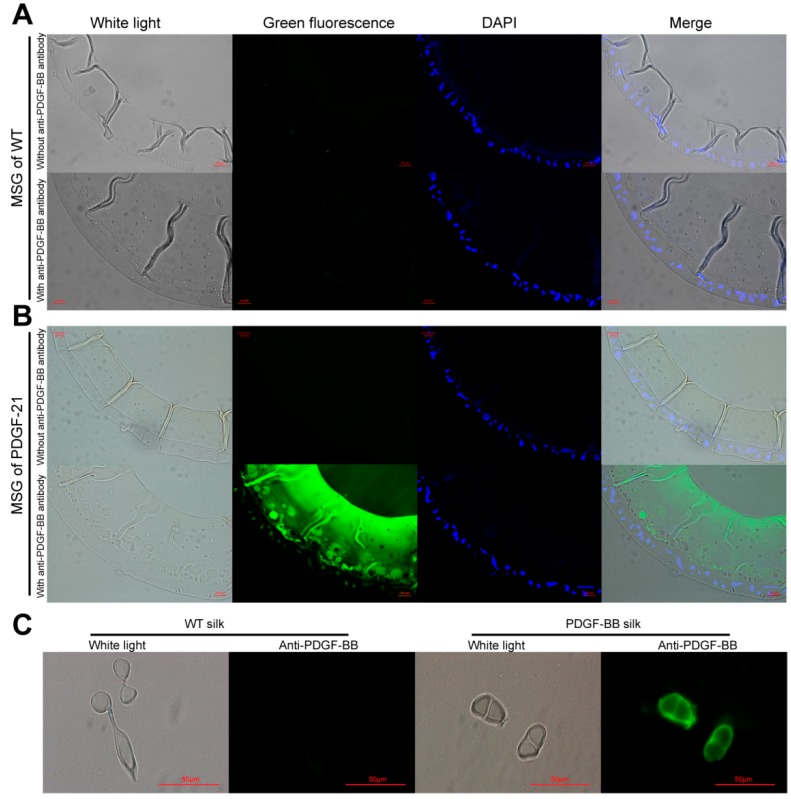
Immunohistochemical analysis of cross-sections of MSG from the WT silkworm (**A**), the transgenic silkworm PDGF-21strain (**B**), and the natural silk fibers from the WT and the transgenic silkworm PDGF-21 strain (**C**). The green fluorescence represents the immunoblot signals of recombinant PDGF-BB proteins; the DAPI stained by blue fluorescence represents the cell nucleus. Scale bar are 50 µm.

The cocoon proteins from the 25 positive silkworm individuals were extracted and analyzed by SDS-PAGE (Figure 2A). The results showed there were significantly visible protein bands from the 25 positive individuals appeared on the CBB stained gel near the 10 kDa protein marker which were distinct to that of wild type. The molecular weight of the distinct protein band was similar to that of the PDGF-BB, which indicated the successful expression of PDGF-BB. In addition, the band intensities of the specific protein from the 25 positive cocoon proteins were different from each other, suggesting that the production levels of PDGF-BB varied dramatically among the different silkworm individuals. Subsequently, the individual PDGF-21 with the highest expression of PDGF-BB was selected and backcrossed with the wild type silkworm to establish the stable transgenic silkworm strain. An inverse PCR analysis showed that the PDGF-21 strain contained a single copy of the *piggyBac* insertion which was located at a nscaf 3003 locus on Chr. 26 (Table 1). The transcription expression of the transgene into the transgenic silkworm was analyzed by RT-PCR and showed significant transcription expression of the *PDGF-B* gene in thePDGF-21 strain compared to the wild type silkworm strain (Figure 2B), and the transcription expression of the *PDGF-B* gene in the PDGF-21 strain competitively decreased that of the endogenous *Sericin-1* gene by 36.3 ± 5.4% (Figure 2C). Subsequently, the extracted proteins from the cocoons of the PDGF-21 strain in both the reduced state and the non-reduced state were analyzed. The results showed that the monomer form of the PDGF-BB protein with a theoretical molecular weight of 14 kDa could be detected in the reduced state, and this was further confirmed by immunoblotting (Figure 2D,E, Lane 4). In addition, the homodimer form of the PDGF-BB protein with a double size molecular weight was detected in the non-reduced state which was also confirmed by immunoblotting (Figure 2D,E, Lane 3).These results strongly indicate that the PDGF-BB recombinant protein synthesized in the middle silk gland of transgenic silkworm could form the PDGF-BB homodimer through intermolecular disulfide bonds which would benefit its bioactivity. The content of the PDGF-BB in the cocoon from the PDGF-21 silkworm strain was estimated to be approximately 0.33 mg/g of the cocoon shell weight (Figure 2F). To visualize the synthesis, secretion and spinning processes of the recombinant PDGF-BB, cross-sections of the MSG and natural silk fiber from the transgenic silkworm were immunohistochemically analyzed, and it was shown that the synthesized PDGF-BB proteins were specifically secreted into the sericin layer of the MSG lumen (Figure 3A,B), and then spun along with the sericins to form the silk thread of the cocoon. Finally, they were distributed in the sericin layer of silk (Figure 3C).

### 2.3. Extraction and Purification of PDGF-BB from the Cocoons of the Transgenic Silkworm

**Figure 4:**
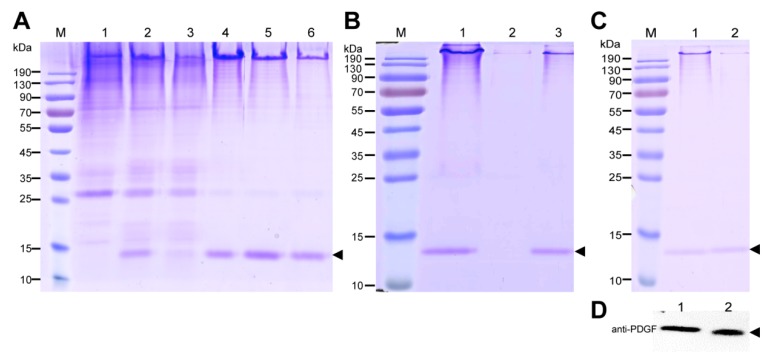
Extraction and purification of recombinant PDGF-BB from the cocoons of the transgenic silkworm PDGF-21 strain. (**A**) SDS-PAGE analysis of the recombinant PDGF-BB in the purification process by a heparin-affinity column. Lane1 represents the total supernatant extracted from the non-transgenic cocoon; Lane 2 represents the total supernatant extracted from of the cocoons of the PDGF-21 transgenic silkworm; Lane 3 represents the constituents flowing through the column. Lanes 4–6 represent the eluted recombinant PDGF-BB with elution buffers containing 100 mM, 150 mM, and 200 mM NaCl, respectively. (**B**) Analysis of the recombinant PDGF-BB in the purification process with a SP-HP column. Lane 1 represents the crude purification of the recombinant PDGF-BB from the heparin-affinity column. Lane 2 represents the constituents flowing through the column. Lane 3 represents the eluted recombinant PDGF-BB with elution buffers containing 100 mM NaCl. (**C**) Analysis of the recombinant PDGF-BB in the purification process by a Q-HP column. Lane1 represents the crude purification of the recombinant PDGF-BB from the SP-HP column; Lane 2 represents the constituents of the recombinant PDGF-BB flowing through the column which were subjected to the SDS-PAGE analysis. (**D**) Quantification of the purified PDGF-BB concentration by Western bolt analysis. Lane 1 represents 1 μL of the purified PDGF-BB. Lane 2 represents 100 ng of the PDGF-BB standard. The concentration of the purified PDGF-BB was estimated to be 100 ng/μL. The black arrowhead points to the recombinant PDGF-BB proteins.

Thirty grams of cocoon powder from the PDGF-21 strain which was estimated to contain 9.9 mg of PDGF-BB was suspended in buffer comprising 8 M urea and 50 mM Tris–HCL of pH 7.0 with a concentration of 30 mg/mL and extracted under a low temperature for 2 days. The concentration of the total extracted proteins from the cocoons was calculated to be 0.108 mg/mL with a BCA Protein Quantification Kit (Beyotine, Shanghai, China).Band intensity quantification on the CBB-stained gel showed that the recombinant PDGF-BB accounted for approximately 8.3% of the total extracted proteins (Figure 4A, Lane 2), which meant approximately 9.0 mg of the recombinant PDGF-BB was extracted from 30 g cocoon powders. The extraction efficiency was estimated to be 90.9%. The extracted sample was then applied to the heparin column. Most of the PDGF-BB was able to specifically bind to the heparin column, and the residual silk proteins were further rinsed from the column (Figure 4A, Lane 3). Following a gradient elution step, the PDGF was eluted from the column by buffers containing a high concentration of NaCl and achieved a purity of 30% (Figure 4A, Lanes 4–6). However, a large proportion of the residual silk protein was also eluted from the heparin column, indicating that endogenous sericins can probably bind to heparin, severely affecting the purification efficiency of PDGF-BB. Thus, the eluted samples shown in Figure 4A; Lanes 5–6 were collected for further processing by ion exchange columns. The pH values of the samples were firstly adjusted to 6.0 so that the recombinant PDGF-BB in the samples was positively charged, while the endogenous sericins were negatively charged under the circumstances. Then, the samples were applied to the cation exchange column where the endogenous sericins flowed past the column, and the recombinant PDGF-BB bound to the column, which achieved an improved purity of the recombinant PDGF-BB of 50% (Figure 4B, Lane 3). Finally, the eluted samples from the cation exchange column were applied to the anion exchange column where the recombinant PDGF-BB flowed past the column and the endogenous sericins bound to the column, which further increased the purity of the recombinant PDGF-BB to 82% (Figure 4C, Lane 2). These results demonstrate that the purification process sufficiently purified the PDGF recombinant protein to apparent homogeneity from the endogenous silk proteins. The obtained recombinant PDGF-BB solution was finally dialyzed against ddH_2_O and adjusted to a concentration of 100ng/μL, inaccordance with the PDGF-BB standard determined by band intensity quantification on the Western blot analysis (Figure 4D). As a result, approximately 4.52 mg of recombinant PDGF-BB was purified from 30 g of cocoons with an estimated recovery rate of 50.2%.

### 2.4. Bioactivity Assays of the Purified PDGF-BB Recombinant Protein

**Figure 5:**
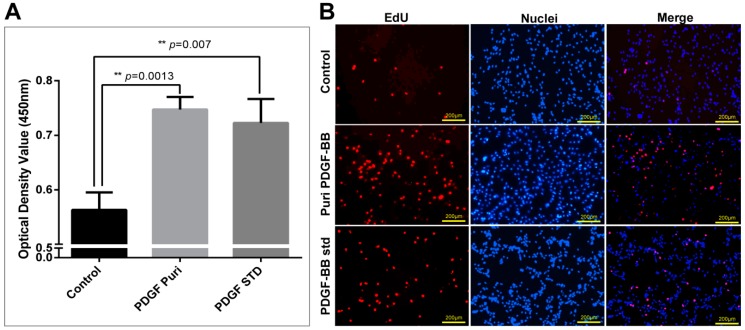
The bioactivity assays of the purified PDGF-BB. (**A**) CCK-8 assay for purified PDGF promoted NIH/3T3 cell proliferation at 24 h after treatment with 100 ng/mL of growth factors. For the significance test: ** *p* < 0.005 vs. control. (**B**) EdU incorporation of NIH/3T3 cells treated by the purified PDGF-BB and an equal amount of the hPDGF-BB standard. Cell nuclei were stained by hoechst 33342 dye (Invitrogen, Carlsbad, CA, USA). Cells undergo proliferation are stained by red fluorescence, cell nucleus are stained by blue fluorescence. The scale bars are 200 µm.

**Figure 6:**
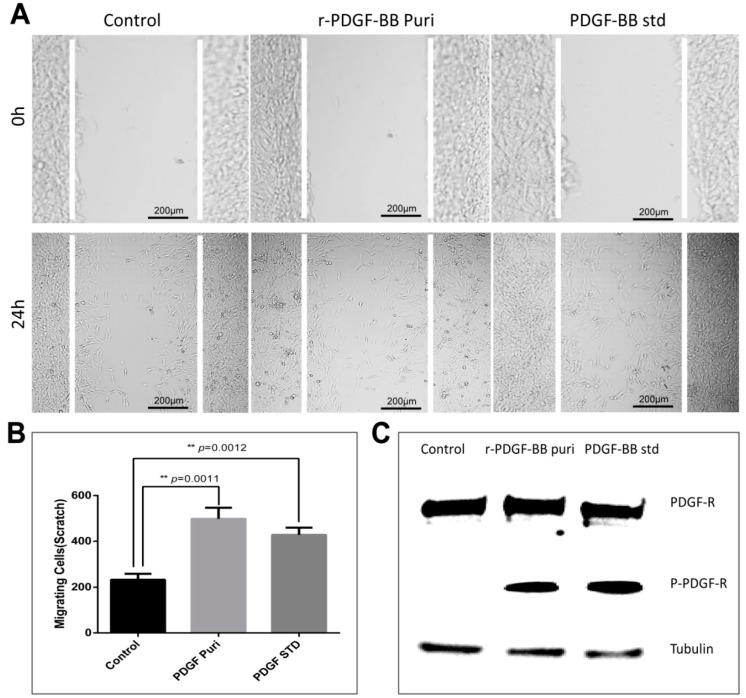
(**A**) In vitro wound healing assay. The scratched NIH/3T3 cells were treated by the purified PDGF-BB and equal amount of hPDGF-BB standard for 24 h. Treatment by an equal amount of PBS was used as control. The scale bars are 200 µm. (**B**) The cell numbers in the scratched areas from different groups after treatment of 24 h. The results are representative of three independent experiments. Asterisks indicate statistical significance based on Student’s *t*-tests (** *p* < 0.01). (**C**) Western blotting to analyze the phosphorylation levels of the PDGF receptor (P-PDGF-R) in NIH/3T3 cells after 24 h treatment with the purified PDGF-BB and an equal amount of the PDGF-BB standard.

The bioactivity of the purified PDGF-BB was estimated by analyzing its effects on cellular proliferation and migration. The starved NIH/3T3 cells were treated by purified PDGF-BB and an equal hPDGF-BB standard was used as a positive control. After cultivation for 24 h, the cell proliferation was measured by CCK-8. The results show that cells treated by the purified PDGF-BB grew well with the typical morphology of 3T3 cellular shape and achieved remarkable absorbance of 0.7473 ± 0.01322 (*n* = 3) at 450 nm which was significantly higher than that of the control group (0.5630 ± 0.01856 (*n* = 3)), and a slightly higher than that of the equal hPDGF-BB standard group (0.7227 ± 0.02530 (*n* = 3)) (Figure 5A). Subsequently, an immunocytochemical analysis by EdU incorporation was used to visualize the proliferation in NIH/3T3 cells (Figure 5B). The results showed that only a small number of NIH/3T3 cells from the control group emitted a weak RFP fluorescence signal; however, more cells treated by purified PDGF-BB exhibited the intensive RFP fluorescence signal which was in accordance with that of the equal commercially purchased hPDGF-BB standard (PDGF-std) group. These results strongly suggest that the purified PDGF recombinant proteins possess strong mitogenic activity to promote the NIH/3T3 cell proliferation. Furthermore, a wound healing assay was performed to investigate the effect of PDGF on promoting NIH/3T3 cell migration. The scratched NIH/3T3 cells treated by purified the PDGF-BB and the equal commercially purchased hPDGF-BB standard (PDGF-std) healed better than the none treated group where were more newly generated cells migrated into the scratch regions (Figure 6A), the migrating cell numbers from the purified PDGF-BB and PDGF-std groups were calculated to be approximate 498.0 ± 28.11 (*n* = 3) and 428.3 ± 18.10 (*n* = 3), respectively, which were significantly higher than that of the control group (232.7 ± 15.17 (*n* = 3)) (Figure 6B). To confirm the recombinant PDGF-BB had activated the molecular signal pathway of the cell proliferation and migration, the phosphorylation level of the PDGF receptor (PDGFR) on the 3T3 cells was examined, the results showed that the phosphorylated PDGFRs from the cells treated by the purified PDGF-BB and PDGF-BB standard could be obviously detected by the immunoblots as opposed to in the control group (Figure 6C), which suggests the purified PDGF-BB from the cocoon of transgenic silkworm could bind to the PDGFRs on the cell membrane to activate the chemotactics, proliferation, and motility of NIH/3T3 cells, and they showed equivalent efficacy in wound healing.

## 3. Discussion

hPDGF-BB has shown therapeutic potential in a wide range of biological processes, such as wound healing [20], vessel remolding [21], and bone repair [3]. The daily increased demands and cost expensive production of hPDGF-BB has aroused interest in large-scale production of recombinant hPDGF-BB in biomedicine and therapeutics.

The silk glands of the silkworm, which was domesticated for over thousand years, possess a huge ability to synthesize an abundant number of silk proteins in a very short period of 5 to 6 days in the fifth instar. Thus, it has been regarded as an desirable bioreactor for producing exogenous proteins of interest in the silk glands of transgenic silkworms [7]. Here, we reported the successful production of recombinant hPDGF-BB in the sericin layer of cocoons by transgenic silkworms. To achieve the adaptative and considerable expression of PDGF-BB in silkworm, the coding sequence of PDGF-BB was optimized according to the silkworm codon usage bias and inserted into the previously-optimized *sericin-1* expression system with potent activity [17]. The construct DNA was microinjected into the silkworm eggs to hereditably integrate into the silkworm genome, with a transformation frequency of 38% which is considerable higher than several previous reports [14,16,22,23,24]. This high transformation frequency was probably caused by the higher activity of the hsp70 promoter used to transiently express the *piggyBac* transposase in the silkworm embryos [25] than that of the commonly used BmAct3 promoter [11], which has been proven and used to achieve higher expression of Cas9 proteins for efficient genome edit in silkworm elsewhere [26].

The PDGF-BB was successfully expressed in the cocoons with a molecular weight accordant to the theory from the positive silkworm individuals. However, their expression levels varied dramatically among the 25 positive silkworm individuals, suggesting the expression of PDGF-BB in different silkworm individuals was severely affected by the chromosome “position affect” caused by the *piggyBac* transposase-mediated random integration into the silkworm genome, which had been reported in several previous studies [12,13,16,27,28]. The PDGF-21 strain with the highest expression of PDGF-BB contained a single copy insertion of *piggyBac* at an intergenic region in the silkworm genome far away from the neighboring endogenous genes. This might help to avoid the effect of chromosome “position affect” on the expression of PDGF-BB. The transcription expression of the *PDGF-B* gene in the PDGF-21 strain competitively decreased that of the endogenous *sericin-1* gene. However, there are no obvious differences in the protein band intensities of sericins from the normal and transgenic silkworm cocoons in SDS-Page. Since the sericins could lubricate the flow of fibroin during spinning processing of silk fiber [29]. If the synthesized sericins proteins decrease severely, it may affect the spinning processing of silk fiber and reduce the silk production. However, we did not observe the abnormal spinning processing of silk fiber in the transgenic silkworm, and the decrease of the economic characteristics of cocoons from the transgenic silkworms. Thus, we speculated that the protein level of sericins was not significantly affected or slightly decreased by the over-expression of PDGF-BB in transgenic silkworm and revealed the potential discordant regulations of the *sericin-1* gene on the transcriptional and translational levels.

The synthesis, secretion, and spinning processes of the recombinant PDGF-BB proteins were visualized, showing that the synthesized PDGF-BB proteins were specifically secreted into the sericin layer of the MSG lumen, and then spun along with the sericins to form the silk thread of cocoon and finally, they were distributed in the sericin layer of silk. This result is consistent with our previous reports [15,30]. The homodimer forms of PDGF-BB in the PDGF-21 strain were analyzed, which strongly indicated that the PDGF-BB recombinant protein synthesized in the middle silk gland of transgenic silkworm could form the homodimer by the intermolecular disulfide bond. Remarkable, the homodimer of the recombinant PDGF-BB was stable even under a harsh extraction conditionof8 M urea and high temperature, which probably benefited from the well protection from the sericin. This phenomenon had also been illustrated by several other literatures which had revealed that the sericin and fibroin in silk fibers might functioned as a stabilizer for the long-term stabilities of the collagen [31], blood components [32], and antibodies [33]. Thus the specific advantage of silk fiber as a stabilizer might be applied for the silk gland based bioreactor for the long-term stability and storage of recombinant proteins in the cocoons, which is lacking in other expression systems such as *E. coli* and yeast.

The purification strategy of recombinant PDGF-BB from the cocoons was constructed according to its heparin-binding property and different isoelectric point to that of the endogenous sericins. Processing comprised extraction, specifically by binding its affinity domain to a heparin-binding column, and purity improvement with a cation exchange column (SP-HP) and anion exchange column (Q-HP). Through the purification process, we were able to purify approximately 4.52 mg of the recombinant PDGF-BB with a purity of 82% from 30 g cocoons of the PDGF-21 strain. The recovery rate was estimated to be 50.2%. During the part of the purification process using the heparin column, we also found that a large proportion of the endogenous sericin proteins could also be eluted from the column, and this severely affected the purification efficiency of recombinant PDGF-BB, suggesting that the sericins from the cocoons of silkworm might be capableof binding specifically to the heparin. Thus, more efforts, such as the introduction of a 6× his tag to PDGF-BB and using Nickel ion affinity chromatography technology for its purification, should be made to optimize the purification strategy and process to achieve better purity of the recombinant PDGF-BB to meet its clinical application in the future. The bioactivity of the purified PDGF-BB was estimated which could successfully phosphorylate the PDGF receptor to activate the molecular signal pathway to achieve the promotion of cell proliferation and migration.

Transgenic silkworms are promising tools for producing recombinant proteins at industrial scales which had been discussed in elsewhere [7]. According to the content of the recombinant PDGF-BB in the cocoons of transgenic silkworm PDGF-21 strain, our calculations showed that it is possible to produce 0.33 kg of PDGF-BB recombinant proteins in a facility with a floor surface of about 500 m^2^ and ten workers rearing a total of about 2.0 million PDGF-21 silkworms in one year. These worms will produce a total of approximately 1000 kg of cocoons, which carry a predicted 0.3 kg of total PDGF-BB recombinant protein yield.

## 4. Materials and Methods

### 4.1. Silkworm Strains and Cell Lines

The silkworm Dazao strain was applied to generate transgenic silkworms. The mouse embryonic fibroblast cell NIH/3T3 cell line was cultivated with Dulbecco’s modified Eagle’s medium (DMEM, Gibco, Waltham, MA, USA) containing 10% (*v*/*v*) fetal bovine serum (FBS, Gibco, Waltham, MA, USA) at 37 °C in a 5% CO_2_ atmosphere.

### 4.2. Transgenic Vector Construction

The coding sequence of the *PDGF-B* gene was optimized according to the silkworm codon bias and synthesized commercially. It was then inserted in the previously constructed *Sericin-1* expression vector pSL1180 [hSer1spDsRedSer1PA] [17] to replace *DsRed* at the *BamH*I and *Not*I sites. Thereafter, the open reading frame (ORF) containing the *PDGF-B* gene was cut by the AscI restriction enzyme and inserted into the framework transgenic vector, pBac [3xp3EGFP, af] [11], cut by the same restrictionenzyme to obtain the final transgenic expression vector, pBac [3xp3EGFP, hSPDGFSer1PA].

### 4.3. Generation of Transgenic Silkworms

The plasmid Mini kit (Qiagen, Hilden, Germany) was used to extract the plasmids for microinjection. After mixture of the pBac [3xp3EGFP, hSPDGFSer1PA] plasmid and the helper plasmid, phsp70PIG [25], which transiently express the *piggyBac* transposase with a 1:1 mole ratio and a final concentration of 500 ng/μL, the mixture plasmid was microinjected (Eppendorf, Hamburg, Germany) into the non-diapause silkworm embryos within 2 h after oviposition, in accordance with a previously reported method [11]. These G0-hatched larvae were fed to oviposit the G1 eggs, which were fluorescently screened at the ocelli and compound eyes where EGFP expresses specifically on the 6th and 7th day using the Olympus SZX12 fluorescence stereomicroscope (Olympus, Tokyo, Japan). The G1 positive individuals were reared to the moth stage and intraspecifically crossed or backcrossed with moths of wild type silkworms (Dazao strain) to generate stable transgenic silkworms.

### 4.4. Genetic Analysis—Inverse PCR

Moth genomic DNA was extracted, and 20 µg was digested with HaeIII at 37 °C overnight and purified using the phenol/chloroform method. Then, 2 µg was circularized by ligation using T4 DNA ligase at 16 °C overnight. The ligated DNA (50–100 ng) was amplified using Taq polymerase under standard conditions with primers (Table 2) designed from the arm region of the *piggyBac* vector. Amplified products were separated using agarose gel electrophoresis and recycled using an OMEGA Gel Extraction Kit (Omega Bio-Tek, Guangzhou, China), and then inserted into the TA-clone vector (TAKARA Bio, Dalian, China) for sequencing. The sequences of transgene insertions were analyzed according to the silkworm genome database: SilkDB (http://www.silkdb.org/silkdb/).

### 4.5. Histological Examination of PDGF-BB Expression in the Cross-Sections of the Middle Silk Gland and Silk Fibers

The middle silk gland (MSG) of 5th instar silkworms at day 6 and the silk fibers were each fixed overnight with 4% (*v*/*v*) formalin and frozen by Tissue-Tek^®^O.C.T.™ compound (Sakura Finetechnical Co., Ltd., Chuo-ku, Japan) before being crosscut into 10 μm thick sections by a freezing microtome. The sections were then immune-blotted with rabbit anti-PDGF polyclonal antibody (BioVision, Zurich, Switzerland) and detected with green fluoresce isothiocyanate-labeled goat anti-rabbit IgG (Beyotime, Shanghai, China) and fluorescence microscopy at the excitation wavelengths of 488 nm for green fluorescent signals (Nikon, Tokyo, Japan).

### 4.6. Transcriptional Expression of PDGF-BB in Transgenic Silkworms by RT-PCR

To detect and compare the expression levels of the exogenous *PDGF-B* and endogenous *sericin-1*, the total RNAs from the middle silk glands of WT and transgenic silkworms on day 6 of 5th instar were extracted using a Total RNA Kit (Omega, Biel/Bienne, Switzerland). 2 μg of each was used for reverse transcription to obtain the corresponding cDNA by a GoScript Reverse Transcription System (Promega, Madison, WI, USA). Subsequently, an equal amount of the cDNA with a concentration of 100 ng/μL was subjected for qRT-PCR assay using the SYBR Premix Ex TaqTM II kit (TaKaRa & Clontech, Dalian, China) on the Applied Biosystems 7500 Fast Real-Time PCR System (Applied Biosystems, Foster City, CA, USA) with a program consisting of an initial denaturing step of 30 s at 95 °C and 40 amplification cycles consisting of 5 s at 95 °C followed by 30 s at 60 °C. The transcriptional expression levels of the exogenous *PDGF-B* or the endogenous *sericin-1* in the WT silkworm and the PDGF-21 transgenic silkworm were firstly normalized to the corresponding internal control SW22934 and then compared with each other. The used primers were shown in Table 2.

### 4.7. Protein Analysis

Cocoons were frozen in liquid nitrogen for one minute and then shattered into powder. The powder with a concentration of 30 mg/mL was suspended into buffer containing 50 mM Tris-HCl and 8 M urea at pH 7.0 at 80 °C for 30 min which could extract the major proteins of the sericin layer from the cocoons. Then, the extracts were centrifuged at 20,000× *g* for 5 min to collect the supernatant samples. The samples, with equal volumes of 20 μL, were separated by the reduced or nonreduced SDS-PAGE (sodium dodecyl sulfate-polyacrylamide gel electrophoresis) with a gel concentration of 12% (*w*/*v*). After electrophoresis, the gels were stained with coomassie brilliant blue (CBB) R250 or transferred to the Western blot analysis. For the Western blot analysis, 10 μL of the protein samples, together with 150 ng and 200 ng of PDGF-BB standard (Abcam, Cambridge, UK) were separated on the polyacrylamide gel and electronically transferred to the polyvinylidene fluoride (PVDF) membrane, followed by the immunoreaction with anti-PDGF antibody (Abcam, Cambridge, UK) at a dilution of 1:10,000 and secondary horse radish peroxidase (HRP)-labeled goat anti-rabbit IgG (Beyotime) at a dilution of 1:20,000. The immunoreacted PVDF membrane was finally visualized using ECL plus (Amersham Biosciences, Little Chalfont, UK), and the images were recorded using a Chemiscope Series (Clinx Science Instruments, Shanghai, China). The content of PDGF-BB in the cocoon shell weight was quantified by densitometric analyzing the intensity of the extracted PDGF-BB with PDFG-BB standard on the Western blots using Image J software (http://rsb.info.nih.gov/ij/).

### 4.8. Extraction and Purification of the Recombinant PDGF-BB from the Cocoon Silk

The cocoons spun by the PDGF-BB transgenic silkworm were ground and extracted with a concentration of 30 mg/mL using the extraction buffer (50 mM Tris-HCl, 8 M urea, pH 7.0) at 4 °C for 2 days. The crude extract was then centrifuged at 20,000× *g* for 20 min, and the supernatant was filtrated with 0.45 μm Durapore^®^PVDF Membrane (Millex^®^-HV). The concentration of the total extracted proteins from the cocoons was calculated with a BCA Protein Quantification Kit (Beyotime, Shanghai, China). The content of the recombinant PDGF-BB protein in the total extracted cocoon proteins was calculated by densitometric analysis of the band intensities of the CBB-stained gel using BandScan5.0 software. Subsequently, the filtered sample was applied to HiTrapTM Heparin HP (GE Healthcare, Chicago, IL, USA) column, and washing steps were conducted using wash buffer (50 mM Tris-HCl, 8 M urea, pH 7.0). The recombinant PDGF-BB was then eluted with elution buffer (50 mM Tris-HCl, 8 M urea, pH 6.0, NaCl with gradient concentrations of 100 mM, 150 mM NaCl, and 200 mM NaCl, respectively). Next, the elution products were applied to a HiTrapTMSP HP (GE Healthcare, Chicago, IL, USA) column, followed by the gradient washing steps using wash buffer (50 mM Tris-HCl, 8 M urea, pH 6.0). The recombinant PDGF was then eluted with elution buffer (50 mM Tris-HCl, 8 M urea, pH 6.0 and NaCl at gradient concentrations of 100 mM and 200 mM, respectively). Finally, the elution products were applied to a HiTrapTM Q HP (GE Healthcare, Chicago, IL, USA) column, and the production flow through was collected and concentrated for SDS-PAGE analysis and concentration calculation by BCA Protein Quantification Kit (Beyotime, Shanghai, China), as described above. The purity of the recombinant PDGF-BB was calculated by densitometric analysis of the bands of purified PDGF-BB on the SDS-PAGE gel using Image J software (http://rsb.info.nih.gov/ij/).

### 4.9. Cell Culture with Purified PDGF-BB

NIH/3T3 cells of 90% confluence were seeded into 96-well plates at a density of 2000 cells per well and starved in 100 µL of DMEM medium containing 0.5% (*w*/*v*) FBS overnight to inactivate cell proliferation. Then, the purified PDGF (100 ng/mL) and an equal amount of hPDGF-BB standard (Abcam, Cambridge, UK) were added to the medium for an additional 24 h.

### 4.10. Cell Proliferation Assays

The NIH/3T3 cells were observed and photographed using a microscope (Nikon, Tokyo, Japan) after being incubated with purified PDGF-BB and hPDGF-BB standard at a dosage of 100 ng/mL for 24 h. Cell proliferation of NIH/3T3 was measured using the Cell Counting Kit-8 (Beyotime, Shanghai, China) and the Click-iT^®^EdU Imaging Kit (Invitrogen, Carlsbad, CA, USA) following the manufacturers’ directions. For the CCK-8 assays, CCK-8 solution (10 µL per well of a 96-well plate) was added to each well of NIH/3T3 cells treated by different groups. The cells were incubated at 37 °C for 1 h, and then the absorbance was measured at 450 nm using a Glomax Multi Detection System (Promega, Madison, WI, USA). Measurements were performed in triplicate and repeated independently three times. For EdU dyeing, after treatment, NIH/3T3 cells were labeled by EdU at a concentration of 2 µM at 37 °C for 2 h. Then, labeled cells were immediately fixed with 3.7% formaldehyde in PBS and permeabilized by 0.5% Triton^®^ X-100, followed by EdU detection using 10 µM Alexa Fluor^®^ 555 azide and imaged by fluorescence microscopy at 555 nm excitation and 565 nm emission maxima. The proliferated cell was photographed under a fluorescence microscope (Olympus).

### 4.11. In Vitro Wound Healing Assay

NIH/3T3 cells were grown in 24-well plates to 100% confluence. Cells were serum-starved overnight and scratched with a sterile pipet tip. Cellular debris was removed by washing with PBS gently. Cells were then stimulated with purified PDGF-BB and the hPDGF-BB standard at a dosage of 100 ng/mL for 24 h. Treatment by an equal amount of PBS was used as control. Initial wounding and the migration of the cells in the scratched area after 24 h were photographically monitored using a microscope (Nikon, Tokyo, Japan). The cell number in the scratched area was calculated using Image J software. Results are representative of three independent experiments. The cells were collected after the same processes, and the total PDGF receptor and phosphorylated PDGF receptor were detected by Western blot analyses using the corresponding antibody (Abcam, Cambridge, UK).

### 4.12. Statistical Data Analysis

Data are means ± standard deviations (SD) for *n =* 3. Statistical analyses were calculated using Student’s *t*-test; * *p* < 0.05, ** *p* < 0.01, and *** *p* < 0.001 was considered statistically significant.

## 5. Conclusions

In summary, this study introduced an available and cost-effective strategy for the production of recombinant PDGF-BB proteins in silk cocoons using a transgenic silkworm. The synthesis, secretion, and spinning of the recombinant PDGF-BB in transgenic silkworm was precisely regulated by our previously established *sericin-1* expression system. The high transcriptional expression of the *PDGF-B* gene competitively inhibited the transcription expression of the endogenous *sericin-1* gene. The synthesized recombinant PDGF-BB formed the homodimer and could be purified from cocoons of the transgenic silkworm. Most importantly, the purified PDGF-BB significantly promoted the growth, proliferation, and migration of NIH/3T3 cells. Our results suggest that the silk gland bioreactor can produce active recombinant PDGF-BB as an efficient mitogen and wound healing agent in silkworm cocoons. This study also provides a reference for the recombinant production of PDGF-BB in other systems.

## Figures and Tables

**Table 1:** The insertion site of transgene in PDGF-21 transgenic silkworm genome.

Strains	Scaffold	Chromosome	Genome Flanking Sequence (5′→3′)
PDGF-21	nscaf 3003:1338	26	AATACGTACTTAA-*piggyBac*-TTAACGTGAAGTA

The target sequence “TTAA” specifically recognized by *piggyBac* transposase was highlighted by red.

**Table 2:** List of oligonucleotides used in this study.

Primers	Sequence (5′→3′)
RT-PDGF-F	TGGCCTGTAAATGCGAAAC
RT-PDGF-R	CGACGTTACCACGACCTTT
RT-Ser1-F	ATCTGAAGACGGTTTCTGGTGGT
RT-Ser1-R	AACTGCCTGAAGTGGTTGTGC
RT-SW22934-F	TTCGTACTGGCTCTTCTCGT
RT-SW22934-R	CAAAGTTGATAGCAATTCCCT
Reverse-pBac-F	TACGCATGATTATCTTTAACGTA
Reverse-pBac-R	GTACTGTCATCTGATGTACCAGG
